# *Ornithodoros faccinii* n. sp. (Acari: Ixodida: Argasidae) parasitizing the frog *Thoropa miliaris* (Amphibia: Anura: Cycloramphidae) in Brazil

**DOI:** 10.1186/s13071-015-0877-3

**Published:** 2015-05-13

**Authors:** Darci Moraes Barros-Battesti, Gabriel Alves Landulfo, Hermes Ribeiro Luz, Arlei Marcili, Valeria Castilho Onofrio, Kátia Maria Famadas

**Affiliations:** Special Laboratory of Zoological Collection, Butantan Institute, São Paulo, SP Brazil; Department of Animal Parasitology, Federal Rural University of Rio de Janeiro, Seropédica, RJ Brazil; Department of Preventive Veterinary Medicine and Animal Science, School of Veterinary Medicine, University of São Paulo, São Paulo, SP Brazil; Postgraduate Course in Veterinary Medicine, University of Santo Amaro, São Paulo, SP Brazil; Center for Natural Sciences and Humanities, Federal University of ABC, São Bernardo do Campo, SP Brazil; Laboratory of Parasitology, Butantan Institute, São Paulo, SP Brazil

**Keywords:** *Ornithodoros*, Larva, Nymph, New species, Soft tick, Frog, Brazil

## Abstract

**Background:**

Most argasid ticks from the Neotropical region are parasites of mammals and birds, with a few records from reptiles. Many species of the genus *Ornithodoros* are known only through larval descriptions, and their chaetotaxy and morphological characteristics have been used to separate the taxa. In the present study, we describe the larva and the nymph of first instar of a new species of the genus *Ornithodoros* that was collected from frogs of the species *Thoropa miliaris*.

**Methods:**

Larvae of *Ornithodoros* were collected from frogs of the species *T. miliaris* at waterfalls in the state of Rio de Janeiro, southeastern Brazil. The larval and nymphal description was based on optical and scanning electron microscopy. Molecular analysis using the argasid 16S rRNA sequences available in GenBank was also conducted.

**Results:**

*Ornithodoros faccinii* sp. n. is closely related to *Ornithodoros clarki* Jones & Clifford, *Ornithodoros marinkellei* Kohls, Clifford & Jones, *Ornithodoros capensis* Neumann and *Ornithodoros sawaii* Kitaoka & Susuki. However, the larval morphology of the new species is unique. The mitochondrial 16S rDNA partial sequence of *O. faccinii* generated in the present study was deposited in GenBank under the number KP861242.

**Conclusions:**

The larvae collected from *Thoropa miliaris* are a new species, *Ornithodoros faccinii* n. sp. This is the first report of argasid ticks on frogs in Brazil, the second on frogs and the third on Amphibia in the Neotropical region.

## Background

Worldwide, there are around 200 argasid species distributed in five genera, among which 87 species are recognized as inhabiting the Neotropical region, and 55 of these belong to the genus *Ornithodoros* [1-4]. Several species of this genus are known only from the larval stage and, therefore, the keys for specific diagnosis refer to this stage [5,6].

The genus *Ornithodoros* is the most diverse of the Argasidae family, and 118 species have been described around the world, of which 16 belong to the Brazilian tick fauna, such as *O. brasiliensis* Aragão, 1923; *O. capensis* Neumann, 1901; *O. cavernicolous* Dantas-Torres, Venzal and Labruna, 2012; *O. fonsecai* (Labruna and Venzal, 2009); *O. hasei* (Schulze, 1935); *O. jul* Schulze, 1940; *O. kohlsi* Guglielmone and Keirans, 2002; *O. marinkellei* Kohls, Clifford & Jones, 1969; *O. mimon* Kohls, Clifford & Jones, 1969; *O. nattereri* Warburton, 1927; *O. rondoniensis* Labruna, Terrassini, Camargo, Brandão, Ribeiro and Estrada-Peña, 2008; *O. rostratus* Aragão, 1911; *O. rudis* Karsh, 1880; *O. setosus* Kohls, Clifford & Jones, 1969; *O. stageri* Cooley & Kohls, 1941; and *O. talaje* (Guérin-Méneville, 1849) [2,3,7-10].

Most argasid ticks in the Neotropical region are parasites of mammals and birds, with a few species on reptiles, such as *Argas transversus* Banks, 1902; *Ornithodoros cyclurae* De La Cruz, 1984; *Ornithodoros darwini* Kohls, Clifford and Hoogstraal, 1969; *Ornithodoros galapagensis* Kohls, Clifford and Hoogstraal, 1969; and *Ornithodoros microlophi* Venzal, Nava and González-Acuña, 2013 [3,11]. There are only three records of parasitism on hosts of the class Amphibia by *Ornithodoros* in this region: larvae of *Ornithodoros* sp. (group *talaje*) recorded on frogs of the species *Eleutherodactylus cooki* Grant, 1932, in Puerto Rico; specimens of *Ornithodoros* reported on toads in Argentina; and *Ornithodoros puertoricensis* Fox, 1947, found on *Rhinella marina* (Linnaeus, 1758) in Panama [12-14].

In the present study, we describe the larva and nymph of first instar of a new species of the genus *Ornithodoros* collected from frogs of the species *Thoropa miliaris* (Spix, 1824) (Anura: Cycloramphidae) and determine the phylogenetic position of this species in comparison with other argasids from different regions. Adults were not described because they were not found in the host’s natural environment and we were unable to obtain this stage under laboratory conditions.

## Methods

### Tick collection and morphological study

Larvae of *Ornithodoros* n. sp. were collected from frogs of the species *T. miliaris*. The frogs were found at the Itinguçu waterfall (22°54’07.47” S, 43°53’34.14” W), which is at the locality of Coroa Grande, municipality of Itaguaí, state of Rio de Janeiro, southeastern Brazil, in 2010 and 2012. Collection of these amphibians was authorized through the System of Authorization and Information on Biodiversity (SISBIO), in accordance with the protocol number 36164–1.

One of the frogs, which was infested with five larvae was also kept in a special vivarium by one of us (H. Luz) until the larvae dropped off. Some engorged larvae dropped off three days later and these specimens were kept in separate vials and were packed into a biological oxygen demand (BOD) incubator at 27° ± 1°C and 90 ± 10% relative humidity (RH) for larval ecdysis. Two nymphs emerged after thirteen days. Eight larvae were prepared on slides (including the holotype) and were measured under a Leica DM2500 microscope coupled to the NiS-Elements BR 64-bit measurement system, v. 3.33.13, and two nymphs were measured under a Leica stereomicroscope. All the measurements were made in micrometers (μm), as the mean followed by the standard deviation and range in parentheses (the holotype measurements are in square brackets). The specimens were prepared and examined, and micrographs were produced through light and scanning electron microscopy (SEM) in the Electron Microscopy Laboratory, at the State University of São Paulo (UNESP), Rio Claro campus. Four larvae and two nymphs were examined under high and low vacuum, respectively.

The description of the new species was based on the literature relating to the taxonomy of the subfamily Ornithodorinae [5,6,15,16]. For the present study, regarding the current usage of valid tick names, we followed the nomenclature proposed in the specific literature on ticks worldwide [2].

### Molecular study

DNA was extracted from two larvae using the guanidine isothiocyanate-phenol technique [17]. The extracted DNA samples were then subjected to PCR targeting a fragment of approximately 460 base pairs (bp) of the mitochondrial 16S rDNA [18]. The products were purified and sequenced using the same primers as used in the PCR. These sequences were aligned using Clustal X [19] and adjusted manually using the GeneDoc software, with sequences previously determined for other argasid species available in GenBank, and also with sequences from *Ixodes holocyclus* Neumann and *Ixodes uriae* White (Ixodidae), which were used as outgroup (the accession numbers of all the sequences are shown in the resulting phylogenetic tree). The phylogenetic tree was inferred by means of the maximum parsimony (MP) method using PAUP version 4.0b10, with 500 replicates of random addition taxa and TBR branch swapping [20]. All positions were equally weighted and Bayesian analysis was performed using MrBayes v3.1.2 with 1,000,000 replicates [21]. The first 25% of the trees represented burn-in, and the remaining trees were used to calculate Bayesian posterior probability.

### Ethical approval

The animals were caught and manipulated in accordance with the recommendations of the Brazilian Institute for the Environment and Renewable Natural Resources - Chico Mendes Institute for Biodiversity Conservation (IBAMA-ICMBio).

## Results

We examined 50 frogs from which 15 were infested, showing prevalence of 30% and the mean intensity of infestation was 1.5 ticks.

### *Ornithodoros faccinii* n. sp. Barros-Battesti, Landulfo & Luz

#### Diagnosis

*Larva* with elongated dorsal plate, almost rectangular; the anterior and posterior margins rounded with sides parallel. Idiosoma dorsal with 10 pairs of dorsolateral setae and 3 pairs of central dorsal setae. Idiosoma ventral with 7 pairs of setae (3 sternal, 3 circumanal and 1 ventrolateral pairs) plus 1 anal pair. Hypostome long, pointed, dental formula 3/3 in the anterior third, then 2/2 posteriorly to the base. Presence of small spurs at the base of hypostome in the lateral position. Trochanter of palpi with spurs in the internal side and ventrally, some of them are bifid. *Nymphs* of first instar with reduced camerostome. Hood and cheeks absent. First pair of posthypostomal setae (Ph1) arising at level of insertion of article II of palpi and half of hypostome. Second pair of posthypostomal setae (Ph2) very short, not reaching the hypostomal basis and corresponding to 1/4 of length of Ph1. Hypostome notched in the apex, dental formula 4/4 apically (3 rows) then 3/3 (3 rows) and 2/2 (3 rows) until posterior margin of article II. Body with mammillae covering both ventral and dorsal surfaces. Genital primordium present. Capsule of Haller’s organ U-shaped.

### Description

#### Larva (Figures 1, 2 and 3)

**Figure 1:**
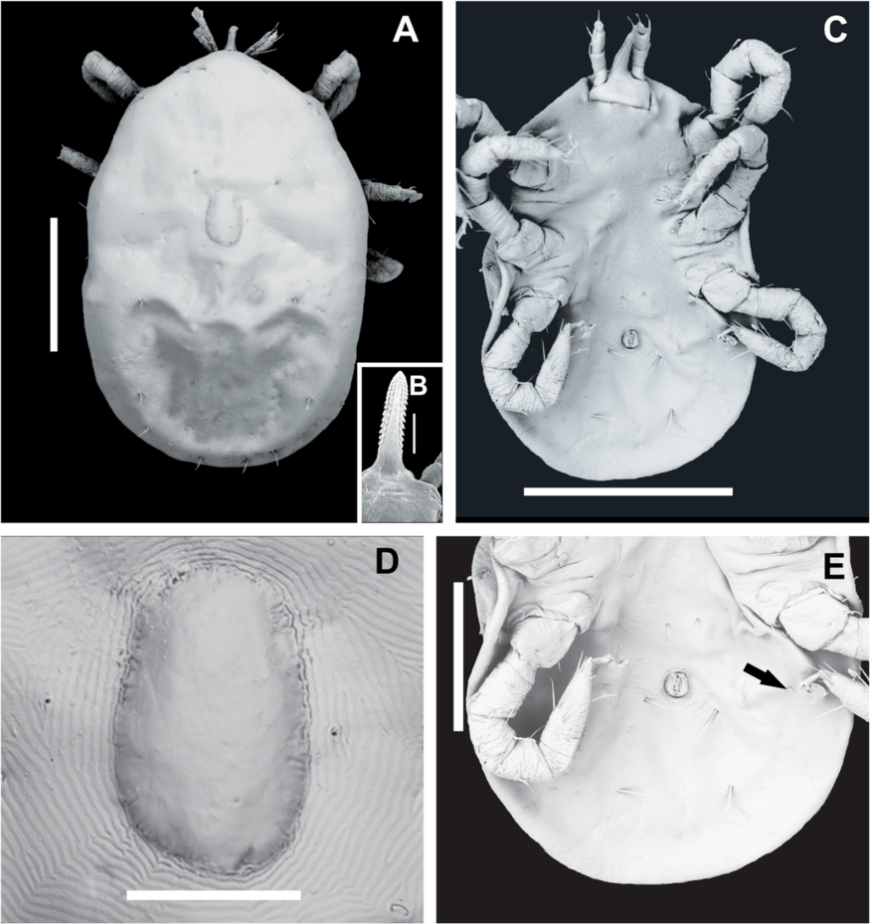
Scanning electron microscopy of idiosoma and capitulum of larvae of *Ornithodoros faccinii* n. sp. **A**. Idiosoma, dorsal view. **B**. Part of basis capituli and hypostome. **C**. Idiosoma, ventral view. **D**. Dorsal plate. **E**. Detail of ventral idiosoma, showing the pair of setae VPL (ventral posterolateral) (black arrow). Scale bars: A. 500 μm; B. 50 μm; C. 500 μm; D. 100 μm; E. 250 μm.

**Figure 2:**
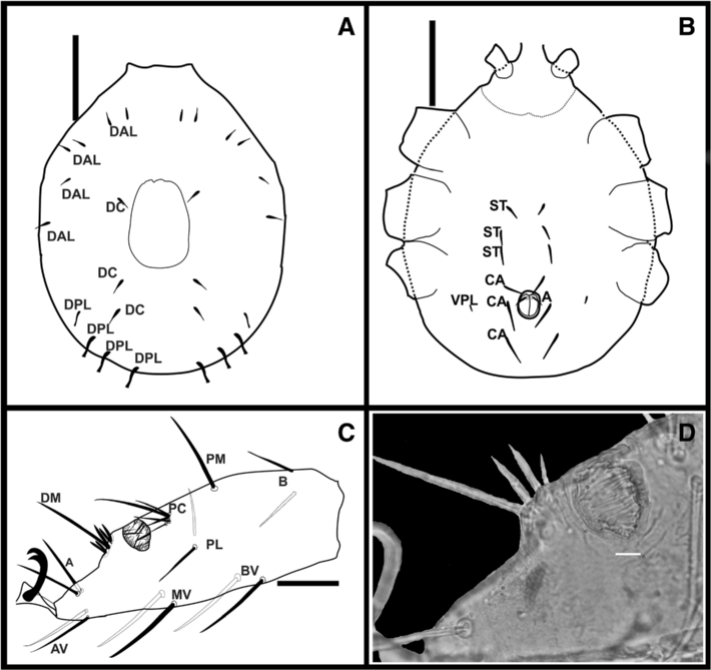
Larvae of *Ornithodoros faccinii* n. sp. **A**. Chaetotaxy of dorsal idiosoma: DAL (dorsal anterolateral setae), DC (dorsal central setae), DPL (dorsal posterolateral setae). **B**. Chaetotaxy of ventral idiosoma: ST (sternal setae), CA (circumanal setae), VPL (ventral posterolateral setae). **C**. Chaetotaxy of tarsus I, A (anterior), DM (dorsomedian), PC (paracapsular), PM (posteromedian), B (basal), AV (anteroventral), MV (midventral), BV (basiventral), PL (posterolateral). **D**. Light micrograph of tarsus I, capsule of Haller’s organ. Scale bars: A and B. 100 μm; C. 50 μm; D. 20 μm.

**Figure 3:**
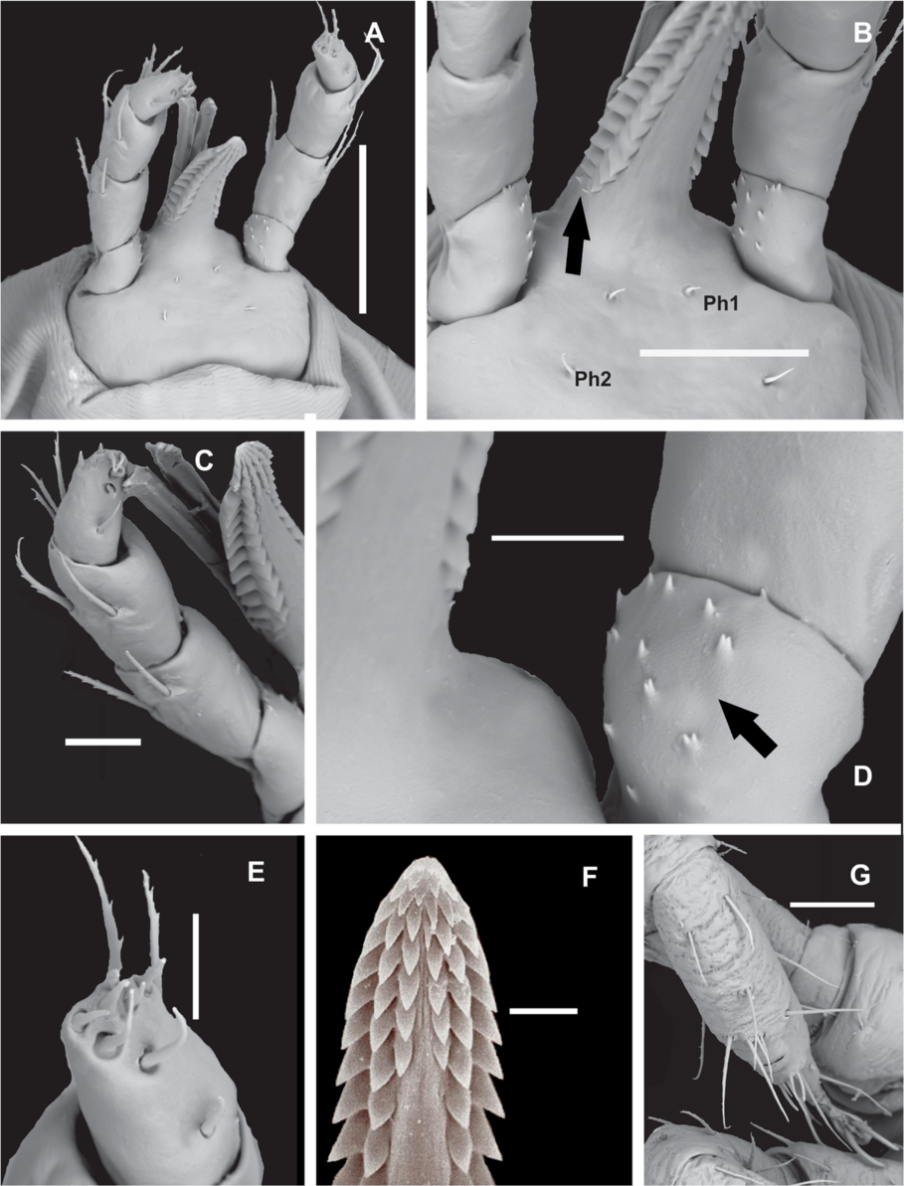
Scanning electron microscopy of gnatosoma and tarsus of larvae of *Ornithodoros faccinii* n. sp. **A**. Capitulum, ventral view. **B**. Details of capitulum showing small spurs at the base of hypostome in the lateral position (black arrow). **C**. Detail of hypostome, palpi and chelicerae. **D**. Trochanter of palpi with 11 short spurs in the inner side, some of them are bifid (black arrow). **E**. Tibiotarsus of palpi. **F**. Hypostome with dental formula 3/3 in the anterior third, and then 2/2 posteriorly to the base. **G**. Tarsi I. Scale bars: A. 100 μm; B. 500 μm; C. 25 μm; D. 15 μm; E. 15 μm; F. 10 μm; G. 50 μm.

*Idiosoma dorsal*: Subcircular (Figures 1A, C, D, 2A-B), broadest near midlength, length 773.32 ± 24.33 (591.12-1220.00) [626.74], width 645.01 ± 15.23 (534.40-901.20) [534.40], with 13 pairs of setae. Dorsal chaetotaxy as follows: 10 dorsolateral (DL) pairs, of which 6 pairs dorsal anterolateral (DAL), length DAL1 30.53 ± 0.35 (30.47-31.26) [30.47], length DAL2 27.24 ± 1.34 (25.87-29.14) [29.14], length DAL3 28.79 ± 0.57 (28.34-29.73) [29.73], length DAL4 32.03 ± 1.53 (31.31-34.86) [32.03], length DAL5 23.00 ± 2.40 (21.10-26.89) [21.10], length DAL6 22.50 ± 6.30 (22.00-35.53) [22.06] (Figure 2A), and 4 pairs dorsal posterolateral (DPL), length DPL1 46.30 ± 1.08 (46.19-48.55) [48.55], length DPL2 43.50 ± 3.34 (41.30-49.23) [49.23], length DPL3 48.66 ± 3.20 (47.04-54.68) [54.68], length DPL4 50.62 ± 3.30 (48.45-56.33) [56.33]; 3 central (DC) pairs, length DC1 33.14 ± 2.65 (32.26-38.28) [38.28], length DC2 24.21 ± 4.02 (23.60-32.42) [32.42], length DC3 29.64 ± 2.46 (27.58-33.53) [33.53]. Dorsal plate smooth, elongated, almost rectangular, with anterior and posterior margins rounded (Figures 1A, D, 2A), length 196.72 ± 0.82 (180.12–201.22) [179.09], width 131.00 ± 0.52 (125.10–133.00) [127.24]. *Idiosoma ventral:* venter with 7 pairs of setae plus 1 anal pair (A). Ventral chaetotaxy (Figures 1C, E, 2B): 3 sternal pairs (ST), length ST1 32.06 ± 2.33 (28.97-34.38) [33.53], length ST2 32.38 ± 3.37 (26.22-35.35) [33.20], length ST3 28.04 ± 2.74 (23.25-30.81) [30.81], 1 pair ventral posterolateral (VPL) and 3 circumanal pairs (CA), length CA1 29.02 ± 0.35 (24.51-34.63) [34.54], length CA2 49.54 ± 0.68 (44.22-63.54) [63.54] and length CA3 59.73 ± 0.24 (55.73-61.43) [58.54]. Posteromedial seta (PMS) absent in most of the larvae (Figure 1E), but present in the holotype.

*Capitulum:* Ventral base with lateral angles slightly rounded. Total length from apex of palpi to base 286.72 ± 13.22 (275.28-307.25) [307.25], width 191.60 ± 8.17 (189.65-207.89) [207.89] (Figure 3A). Two pairs of posthypostomal setae: Ph_1_ length 6.04 ± 0.11 (5.53-6.63) [5.53], Ph_2_ length 10.2 ± 0.28 (9.02-10.23) [9.02]; distance between Ph_1_ setae 28.32 ± 0.56 (27.72-34.92) [34.92], and between Ph_2_ setae 59.10 ± 0.21 (57.60-66.75) [66.75] (Figure 3B). *Hypostome:* long, pointed, small spurs at the base of hypostome in the lateral position (Figures 1B, 3B), dental formula 3/3 in the anterior third, then 2/2 posteriorly to the base (Figures 1B, 3C, F), length (in 3 specimens only) from apex to Ph_1_ setae 194.33 ± 4.80 (188.66-200.50) [194.33], width in medial portion 48.83 ± 4.90 (47.77–58.64) [58.64]. *Palpi:* length 195.47 ± 5.15 (187.92-200.71) [198.84], length of palpal articles I–IV (Trochanter, Femur, Genu, Tibiotarsus), respectively: 39.16 ± 7.50 (32.94-51.70) [43.61], 53.60 ± 0.31 (51.13-57.91) [56.00], 58.99 ± 2.90 (53.83-61.80) [61.80] and 35.71 ± 0.52 (34.23-66.45) [40.60]; number of setae on palpal articles I-IV is 0, 4 (1V, 3D), 5 (1V, 4D) and 9 tibiotarsal, respectively (Figures 3A, C, E). There are 11 short spurs in the inner side in each trochanter, some of them are bifid (Figures 3B, D). These spurs can also be observed by optical microscopy using immersion objective.

*Legs:* Tarsus I (Figures 2C, 3G) length (including claw) 325.47 ± 16.99 (318.08-362.84) [318.08], width 91.35 ± 0.89 (89.94-92.26) [92.26]. Setal abbreviation of Tarsus I – 1 pair apical (A), 1 pair basal (B), 1 seta distomedian (DM), 1 seta posteromedian (PM), 1 pair apicoventral (AV), 1 pair midventral (MV), 1 pair basiventral (BV), 1 pair posterolateral (PL) and 5 setae paracapsular (PC); 5 small setae together with DM seta. Capsule of Haller’s organ placed centrally, opening as transverse slit, without reticulations inside, but with at least 6 larger setae and many other smaller setae in the marginal border (Figure 2D).

### First nymphal instar (N1) (Figure 4)

**Figure 4:**
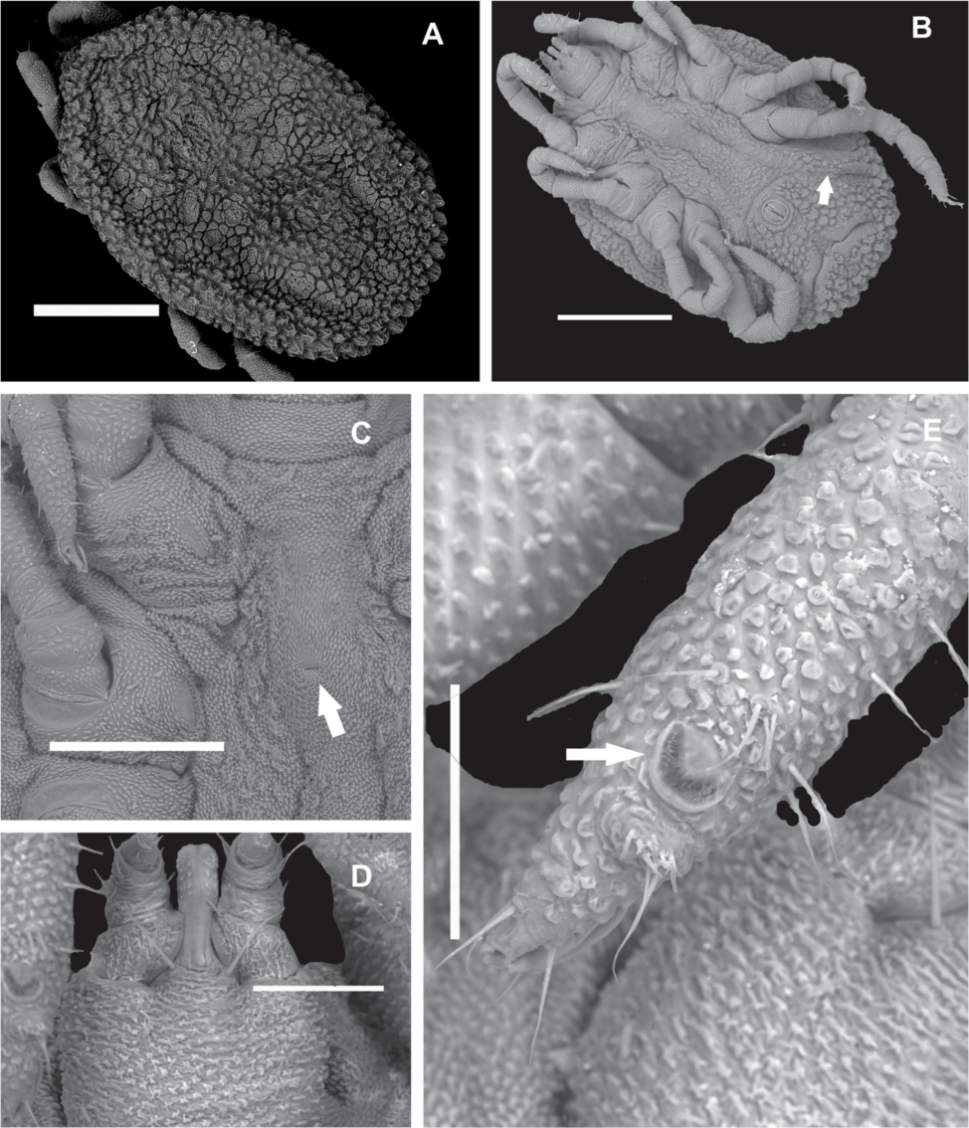
Scanning electron microscopy of nymphs of *Ornithodoros faccinii* n. sp. **A**. Idiosoma, dorsal view. **B**. Idiosoma, ventral view, showing the preanal groove reaching the sides of the body (white arrow). **C**. Genital primordium (white arrow) on the ventral idiosoma. **D**. Capitulum. **E**. Tarsi I and U-shaped capsule (white arrow), partially covered by a V-shaped membrane. Scale bars: A. 500 μm; B. 500 μm; C. 250 μm; D. 100 μm; E. 100 μm.

*Idiosoma dorsal:* Body shape oval, slightly pointed anteriorly, sides nearly parallel, length 1589.43 ± 231.60 (1425.67-1753.20), width 1049.89 ± 191.60 (914.41-1185.37). Mammillae with irregular surface and size throughout dorsum, larger at the lateral and posterior margin; short hairs present, discs placed in large depressed areas within marginal grooves. Disc size and shape variable, as follows: lateral area – 2 anterolateral pairs of small rounded discs, contiguous, at the level of legs II, and 1 small slightly elongated laterocentral pair; central area – 3 small rounded discs placed at the level of legs III; and posterior area – 3 elongated discs located posteriorly to 4th pair of legs. Submarginal grooves distinct and fused anteriorly (Figure 4A). *Idiosoma ventral:* Mammillae present on entire surface, larger at posterior margin. Aperture of coxal glands posterior to coxae I. Genital primordium between coxae II (Figure 4B-C). Preanal, transverse postanal and medial postanal grooves present. Dorsoventral groove absent. Preanal groove reaching the sides of the body; supracoxal and coxal folds present. Anal plate elliptical. Spiracular plate semicircular, placed between coxae III-IV, length 80.94 ± 2.77 (78.98-82.91) at maximal diameter.

*Capitulum:* Ventral base slightly wider than long, micromammillated, length 237.47 ± 1.38 (236.49-238.45), width 189.79 ± 11.02 (182.00-197.59). Basal capituli with 1 pair of posterolateral short setae (Figure 4D). Hood and cheeks absent; camerostome rudimentary. First pair of posthypostomal setae (Ph1) exceeding level of insertion of article II of palpi and half of hypostome. Second pair of posthypostomal setae (Ph2) short, not exceeding hypostomal base and corresponding to 1/4 of length of Ph1. Palpi moderate in size, with few setae; articles micromammillated with ventromedial integumental ridge-like extension in internal margin of article I, which covers part of hypostomal basis, reaching half of the hypostome; length article I 38.73 ± 5.93 (34.54-42.93), article II 36.89 ± 5.94 (32.69-41.10), article III 37.71 ± 2.26 (36.11-39.31) and article IV 25.69 ± 0.43 (25.38-26.00). *Hypostome:* with slightly notched apex, dentition 4/4 apically (only two rows defined) then 3/3 (two rows defined), and then 2/2 until close to posterior margin of article II, length from apex to Ph1 97.95 ± 0.48 (97.61-98.29) (Figure 4D).

*Legs:* With surface micromammillated; coxae I-IV with various mammillae (Figure 4E); small setae sparsely distributed throughout articles. Coxa I well separated from coxa II by an intercoxal fold (Figure 4B-C), coxae II–IV contiguous. Tarsus I with short and long setae, mainly near to Haller’s organ on the subapical dorsal protuberance, dorsal humps absent, pulvilli reduced. Tarsi with claws, lengths of tarsus I and IV, respectively, 310.63 ± 13.67 (300.96-320.30) and 365.25 ± 7.56 (359.90-370.60). Capsule of Haller’s organ U-shaped, partially covered by a membrane, with several setae internally (Figure 4E).

### Phylogenetic position

**Figure 5:**
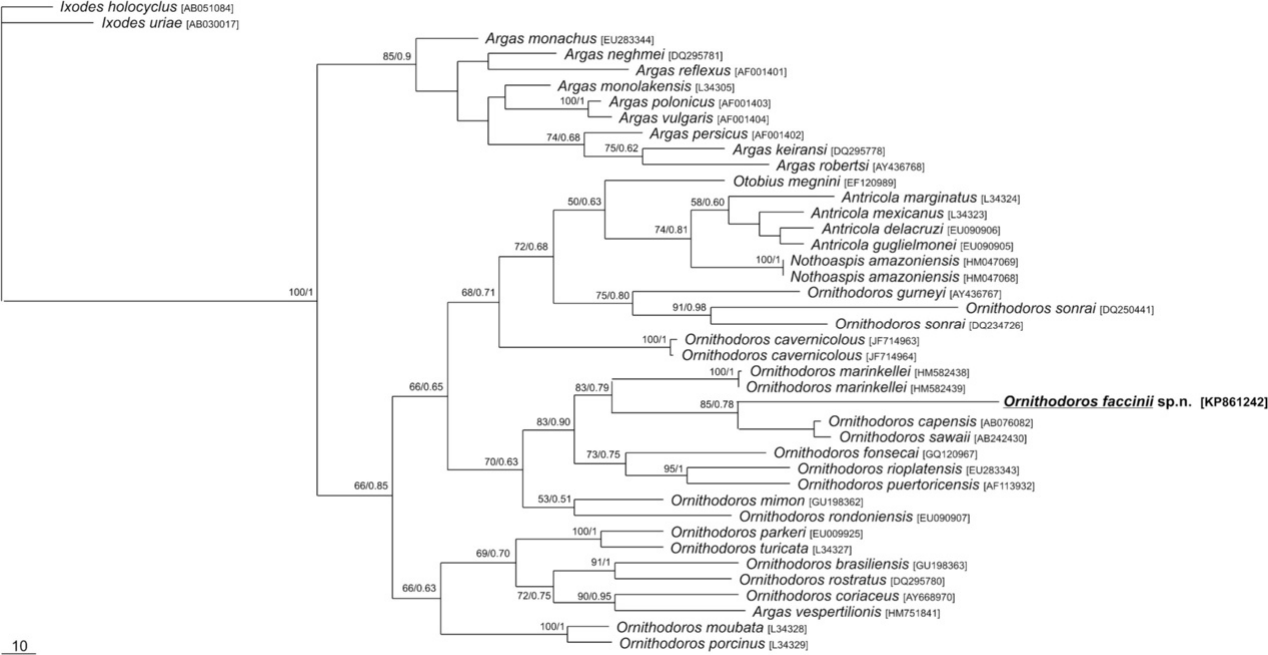
Phylogenetic tree based on the 16S rDNA ticks. The alignment was produced using Clustal X and the tree was inferred by means of the MP method with 500 replicates of random addition taxa. The species *Ixodes holocyclus* and *Ixodes uriae* were used as outgroup. The Bayesian support (posterior probability) values are derived from 1,000,000 replicates.

The phylogenetic relationships based on a partial sequence of the mitochondrial 16S rDNA gene (Figure 5) grouped *O. faccinii* n. sp. with *O. capensis* and *Ornithodoros sawaii* Kitaoka & Susuki, 1973, within a strongly supported branch (85% bootstrap and 0.78 posterior probability)*.* The sequence divergence between *O. faccinii* n. sp. and *O. capensis* was 19.8% and between *O. faccinii* n. sp. and *O. sawaii*, 22%*.* The larva of the new species is also related to larvae of *O. marinkellei* and *O. fonsecai*.

### Taxonomic summary

Order Ixodida Lech, 1815

Family Argasidae Canestrini, 1890

Genus *Ornithodoros* Koch, 1844

Species *Ornithodoros faccinii* n. sp. Barros-Battesti, Landulfo & Luz

### Type-host

*Thoropa miliaris* (Spix, 1824) (Amphibia: Anura: Cycloramphidae).

### Type-locality

Itinguçú waterfall (22°54’07.47” S, 43°53’34.14” W), which is at the locality of Coroa Grande, municipality of Itaguaí, state of Rio de Janeiro, Brazil.

### Collectors and date of collection

H. R. Luz and G. A. Landulfo, November 26, 2010.

### Type material

Holotype larva mounted on slide deposited in the Acari Collection of the Butantan Institute, under the number (IBSP 10316); 7 paratype larvae in slides, 5 paratype larvae and 2 paratype nymphs in 80% ethanol (IBSP 10317); 2 paratype larvae deposited in the National Tick Collection (Coleção Nacional de Carrapatos, CNC) of the School of Veterinary Medicine, University of São Paulo, São Paulo, Brazil (CNC 3002); 2 paratype larvae deposited in the tick collection of the National Institute of Agricultural and Livestock Technology (Instituto Nacional de Tecnología Agropecuaria, INTA), Rafaela, Santa Fé, Argentina (INTA 2285); 2 paratype larvae deposited in the tick collection of the Department of Veterinary Parasitology (DPVURU), Veterinary School, University of the Republic, CENUR Litoral Norte, Salto, Uruguay (DPVURU 877); and 2 paratype larvae deposited in the U.S. National Tick Collection, Georgia, Southern University, Statesboro (USNMENT 00862006). They all come from same host and locality.

### Gene sequences

The mitochondrial 16S rDNA partial sequence of *O. faccinii* n. sp. generated in the present study was deposited in GenBank under the number KP861242.

### Etymology

The name of the species is a tribute to Professor Dr. João Luiz Horácio Faccini of the Federal Rural University of Rio de Janeiro, who has always been devoted to studies of parasitic mites and ticks in Brazil.

### General

In accordance with section 8.5 of the International Code of Zoological Nomenclature (ICZN), details of the new species have been submitted to ZooBank with the life science identifier (LSID) zoobank.org:pub: A229A69C-9DD5-4E93-8 AC8-8DF237556CA8.

## Discussion

### Species relationships

The larvae of *O. faccinii* n. sp. have morphological characteristics that indicate that this species does not belong to any subgenus previously proposed, although they present some characteristics close to the subgenus *Alectorobius*. The larvae of this subgenus present dorsal plate pyriform and widest posteriorly; dorsal surface with 11–18 pairs of dorsolateral setae and 3–5 pairs of central setae, usually pointed and barbed; ventral surface with 8 or 9 pairs of setae plus a posteromedial seta; basal capituli without cornua or auriculae and palpal articles without spines [15]. Nevertheless, the presence of small spurs at the lateral base of the hypostome and at the inner side of the trochanter of the palpi, as well as the dorsal plate that is smooth, elongated almost rectangular, with the anterior margin slightly rounded and narrowed and the posterior rounded and almost convex, comprise the main characteristics that morphologically distinguish *O. faccinii* n. sp. from the other species of *Alectorobius*. Although the new species is phylogenetically related to *O. capensis* [5], occurring in the Nearctic, Ethiopic, Oriental and Palearctic regions, and *O. sawaii* [22]*,* restricted to the Japanese islands, which are both parasites of marine birds, it is morphologically distant. The larvae of *O. capensis* and *O. sawaii*, present high numbers of dorsal setae (22–25 pairs), dorsal plate pyriform, hypostome blunt and presence of PMS setae. The reduced number of dorsal setae (around 13), and the hypostome, which is pointed with denticles throughout its entire length in *O. faccinii* n. sp., resemble *O. fonsecai* (13–14 pairs of dorsal setae) and *O. marinkellei* (13 pairs of dorsal setae) (*Subparmatus*).

Nevertheless, the dorsal plate, which is triangular in this last species, and pyriform in *O. fonsecai*, distinguish them from *O. faccinii* n. sp. [23-25]. Regarding the dorsal plate, *Ornithodoros clarki* Jones & Clifford, 1972 is in fact similar to *O. faccinii* n. sp., however the dorsal plate in *O. clarki* is very big (320x200 μm) and almost straight in the posterior margin [16], while in *O. faccinii* n. sp. it is smaller (197x131 μm), and almost convex in the posterior margin. Besides, *O. clarki* does not have spurs at the lateral base of the hypostome and at the inner side of the trochanter of the palpi.

Few dorsal setae are also common in *O. brasiliensis* and *O. rostratus*, which in turn have dorsal plate similar to *O. faccinii* n. sp. [4]. But the new species is easily separated from them by the presence of long and pointed hypostomes.

The PMS (absent in most larvae of *O. faccinii* n. sp.) is also absent in *O. marinkellei* [25], in *O. setosus* [6], *O. amblus* [26], *O. clarki* [16], *O. spheniscus* [27], and *O. yunkeri* [28]*.* However, *O. marinkellei* presents 2 large auricula-like projections in the capitulum, which are absent in the new species [25] and in others species that do not belong to the *Subparmatus* subgenus. The species *O. setosus*, *O. amblus*, *O. spheniscus* and *O. yunkeri* present the dorsal plate typically pyriform that distinguish them from *O. faccinii* n. sp.

Except for *O. mimon* that has all nymphal instars described with details, among all other 46 species of *Ornithodoros* belonging to the *Alectorobius* subgenus of the Neotropics, only 21 of them have a brief description of an unspecified nymphal instar.

The nymph of *O. faccinii* n. sp. has characteristics that resemble species of *Alectorobius*, such as integument with distinct mammillae and discs, idiosoma pointed anteriorly, legs with micromammillated cuticle and absence of dorsal humps on tarsi I-IV [15]. However, the absence of hood and cheeks, and the U-shaped capsule of Haller’s organ separate the new species from most other species of the genus *Ornithodoros* belonging to the group *Alectorobius* in the Neotropical region, for which the nymphal stage has been described [29]. The U-shaped capsule aperture of Haller’s organ seems to be unique to *O. faccinii* n. sp., as far as we are currently aware. Nymphs of the species *Ornithodoros azteci* Mathenson 1935, *O. capensis*, *O. cavenicolous*, *Ornithodoros dyeri* Cooley & Kohls 1940, and *Ornithodoros yumatensis* Cooley & Kohls 1941 share the absence of a hood with *O. faccinii* n. sp. However, the presence of cheeks in the nymphs of those previously described species distinguishes them from the nymphs of the new species. *Ornithodoros stageri* Cooley & Kohls, 1941, also has no hood and cheeks, but the legs of this species have a smooth surface while the legs of *O. faccinii* n. sp. are micromammillated. An incomplete description of nymphs of *O. sawaii* has been presented and the authors commented that the nymphs and adults are quite similar [22]. Unfortunately, most descriptions of nymphs of the genus *Ornithodoros* are generally poor on detail and illustrations, and this inhibits the ability to make comparisons between species [29].

The presence of genital primordium in the N1 of *O. faccinii* n. sp. may indicate that this species only has one nymphal instar, because the genital primordium is indicative that the next stage could be the adult [30]. *Otobius lagophilus* Cooley & Kohls, 1940, and *Ornithodoros peropteryx* Kohls *et al.*, 1969, are species of soft ticks that only have one nymphal stage and thus, these species undergo two molts in the process of reaching the adult stage [31,32]. According to southern specialists, it is possible there are more species with this behavior, especially in the Neotropical region [32]. Although it has been reported that the nymphs of *O. sawaii* present a smooth circular structure with very small setae ventrally, in place of the genital aperture, there was no mention of which nymphal instar was used in the description [22].

The host *Thoropa miliaris* is a frog that is endemic to the Atlantic Forest and is found in rocky environments near the coast in southeastern Brazil [33]. Thus, this report provides the first record of an argasid tick parasitizing Amphibia in Brazil.

The morphological and phylogenetic studies are congruent and they support *O. faccinii* as a new species, thereby increasing the Brazilian argasid fauna to 22 species.

## Conclusions

The morphological and phylogenetic evidence from the *Ornithodoros* larvae collected from *T. miliaris* made it possible to describe a new species named *Ornithodoros faccinii*. This is the first report of argasid ticks on frogs in Brazil, the second report on frogs and the third on Amphibia in the Neotropical region. Moreover, this report expands the number of species of the genus *Ornithodoros* for this region.
